# Supplementary material for: The Aerogen® Solo Is an Alternative to the Small Particle Aerosol Generator (SPAG-2) for Administration of Inhaled Ribavirin
Source: Pharmaceutics. 2020 Nov 29;12(12):1163. doi: 10.3390/pharmaceutics12121163 (PMC7760766; doi:10.3390/pharmaceutics12121163)
Supplement: Supplementary file 1 [file pharmaceutics-12-01163-s001.pdf]

St. Jude Children's Research Hospital
ALSAC • Danny Thomas, Founder
Finding cures. Saving children.

Respiratory Therapy
POLICY & PROCEDURE MANUAL

Date of Original: 11/15/18
Date of Review:
Date of Revision: 9/4/19

RIBAVIRIN ADMINISTRATION (Aerogen nebulizer)

PURPOSE:
1. **Standardize the administration and delivery of ribavirin by aerosol**
2. **To protect the patient and caregivers by standardizing precautions for ribavirin delivery**

POLICY:

The patient room must be set up according to St Jude's Environmental Health and Safety policy prior to the administration of Ribavirin. Room preparation is to be done by the nursing staff. *See procedure section below
https://home.stjude.org/ehs/Pages/ribavirin-precautions.aspx

- Negative pressure room is mandatory for Ribavirin administration.
- Preparation of the room is the responsibility of the nursing staff.
- Ribavirin will be delivered from pharmacy in primed Aerogen syringe set appropriate for use on the MedFusion 3500 syringe pump.
- The syringe pumps will be used, maintained, and stored by the respiratory therapy department.
- The PPE cart is the shared responsibility of the respiratory therapist who administers the first dose of ribavirin and the nursing coordinator for that area.
- Proper sign including contact precautions and STOP will be shared responsibility of the nursing and respiratory care.
- All instances of spilled or leaked ribavirin must be documented in the event reporting system.
- Ribavirin may be delivered to a patient during mechanical ventilation in a very specific set of circumstances. The final decision on the use of ribavirin is at the discretion of the ICU physician on service.

EQUIPMENT:
- Negative pressure room
- DeMistifier 2000 HEPA filtration system.
- DeMistifier negative pressure bed enclosure with canopy.
- MedFusion 3500 Syringe pump
- Flowmeter
- Aerogen vibrating mesh nebulizer
- Delivery Supplies (O2 tubing, O2 reducer, large bore tubing, T adaptor, and aerosol face mask)
- Prescribed medication
- STOP sign for the door during drug administration

PPE

- N95 or Positive Air Pressure mask required
- Chemo resistant Gown
- Double Gloves
- Shoe covers

PROCEDURE:

1. Verify physician order in the medical record.
2. Verify the negative pressure room is working properly
3. Request medication from pharmacy.
4. Set up the Demistifier 2000 HEPA filtration system and Demistifier negative pressure bed enclosure with canopy around the patient bed.
5. Place MedFusion 3500 syringe pump in patient room.
6. Explain procedure to the patient and family answering any questions the patient or family may have.
7. Don appropriate PPE for strict isolation (See PPE above)
8. Confirm patient identity by inspecting patient arm band.
9. Scan medication into the medical record eMAR.
10. Assemble Aerogen vibrating mesh nebulizer for medication delivery.
11. Assess the patient.
12. Turn on the Demistifier before beginning the aerosol.
13. Program syringe pump to deliver medication
14. Assemble delivery supplies attaching one end of the O2 tubing to the flow meter and the other end to the large oxygen tubing and T Adaptor.
15. Set the flow on the flow meter to $\geq$ 8 liters per minute.
16. Begin medication delivery on syringe pump at a rate of 14 ml/hr
17. Begin aerosol therapy by hitting the blue button on the aerogen nebulizer and holding the button for 5 seconds to activate continuous nebulizer mode.
18. Observe the patient for signs of respiratory distress. If signs of respiratory distress are noticed
    - Discontinue aerosol immediately
    - Assist patient with any medical interventions required.
    - Notify the ordering physician of the patient’s reaction to the therapy.
19. Document all care in the medical record.
20. Reassess patient every 2 hours while aerosol is running alternating every 1 hour with the patient’s nurse.
21. At the end of each prescribed treatment time:
    - Turn off the Aerogen nebulizer at the end of prescribed treatment run time.
    - Turn off the Demistifier.
    - Document all care in the medical record.
    - Place any drug remaining in the nebulizer or IV tubing into a red bag and remove to the soiled utility closet.
    - Place large bore tubing, T adaptor, aerosol mask and O2 reducer into a red bag and remove to soiled utility closet.
22. After the last prescribed treatment:
    - Place the Demistifier canopy and aerosol tubing and mask into a red bag and remove to soiled utility closet.
    - Clean the Demistifier and bed enclosure with Antimicrobial wipes and return to the respiratory care department.
    - Clean the MedFusion 3500 syringe pump with Antimicrobial wipes and return to the respiratory care department.

RIBAVIRIN WITH MECHANICAL VENTILATION

Ribavirin may be delivered to a patient during mechanical ventilation in a very specific set of circumstances. The patient must meet all criteria for ribavirin as set forth by Office of Infectious Disease. In addition to these criteria:

1. The patient's viral infection must be, in the agreement of the care team, the primary reason or a major contributor to the need for mechanical ventilation without contributing comorbidities.
2. The patient's endotracheal tube size must be 4.0 or larger.
3. The final decision on the use of ribavirin is at the sole discretion of the ICU physician on service.

PROCEDURE

All procedure steps and precautions are the same as listed above with the following exceptions and additions:

1. Because the ventilator has a closed, filtered circuit; the HEPA filtration system and canopy enclosure is not needed when delivery ribavirin with mechanical ventilation.
2. Double filters will be used on both inspiratory and expiratory limbs of the ventilator circuit during ribavirin administration.
3. The patency of the endotracheal tube and stability of exhaled tidal volumes and pressures will be checked Q30min by the respiratory therapist and documented in the medical record. Any change in ventilation mechanics will prompt the respiratory therapist to change the filter closest to the patient.
4. After completing the ribavirin treatment, all filters will be changed, with the used filters placed in red biohazard bags and removed to the soiled utility closet.

Environmental Health and Safety policy:

- Individuals that are pregnant must not provide care to patients receiving Ribavirin.
- Individuals that must enter the patient room during Ribavirin treatment (e.g. essential caregivers, patient's family, visitors, etc.) must wear the PPE described below.
  - A double layer of nitrile chemotherapy resistant gloves (the outer glove to be changed as often as necessary. Hands to be washed after gloves are doffed)
  - Nurses must wear a Powered Air-Purifying Respirator (PAPR). Other caregivers must wear an N95 respirator and safety glasses or PAPR respirator.
  - Chemotherapy resistant gown/apron in lieu of regular isolation precaution gown.
  - Shoe covers. Shoe covers must be donned before entering the room and doffed while crossing the threshold from the patient room into the anteroom.
- Drape the horizontal surfaces on furnishings (e.g. cabinets, tables, etc.) with towels, sheets, or disposable draping material. These drapes should remain in place for at least 1 hour after administration has been completed and then removed as soiled linen or, in the case of disposable materials, discarded as yellow bag waste. Gloves must be worn while handling these items.
- Shoe covers must be worn by all staff members entering the patient room. They must be removed while crossing the threshold from the patient room to the ante room to prevent the spread of Ribavirin contamination that may have been picked up from the floor of the patient room.
- Individuals entering the patient room must continue wearing a respirator and PPE for at least one hour after Ribavirin treatment has been discontinued. This will allow time for the Ribavirin aerosol to settle out of the air. Shoe covers must be worn until after EVS has wiped down floors with bleach wipes.

NOTE: When needed, make appropriate referrals: Child Life, Rehab, Nutrition, etc. according to assessed patient need.

Other Resources:
https://msdsmanagement.msdsonline.com/90430c1c-d8a1-477c-9bcb-9637a5876716/pdf/?libraryID=REI809&pageID=3&nw=true&autoOpen=false&userDocumentQueueID=0

https://home.stjude.org/nursing/Policies/ribavirin-administration.pdf#search=Ribavirin

Revision History

Revision Number/ Last Revision	Change Initiated By	Date	Policy Number	Title	Revision Description

Related Policies:

Policy Source	Policy Number	Title

Approved by: ___________________________ ________________________
Other Department Head – when needed Date

Approved by: ___________________________ ________________________
Director, Critical Care-Respiratory Therapy Date

Approved by: ___________________________ ________________________
Director, Critical Care Division Date

Approved by: ___________________________ ________________________
V.P., Patient Care Services Date

Supplementary Table 1. Advantages and disadvantages of the Small-Particle Aerosol Generator delivery system (SPAG-2) and the Aerogen® Solo

	SPAG-2	Aerogen® Solo
Advantages	FDA approved for administration of aerosolized ribavirin.<br><br>Allows multiple drug delivery concentrations and delivery times.	Can be used multiple times without replacing the disposables.<br><br>Can deliver ribavirin to patient using oxygen or room air.<br><br>No crystallization was observed on the floor or patient bed. Crystals remain in the short length of large bore tubing, the aerosol mask, and on the skin around the mouth and nose.<br><br>Can be contained to a single IV pole during and between treatments. The disposables may be placed in a labeled specimen zip lock bag and hung on the IV pole between use.<br><br>Parts are all disposable and replaceable.
Disadvantages	Limited supply available from manufacturer.<br><br>Parts are unavailable for replacement from manufacturer.<br><br>Device is large and unwieldy, requires separate table or stand, takes up space in crowded room.<br><br>Set-up requires 6ft or more of large bore tubing and an aerosol mask.<br><br>Disposable tubing and mask have to be removed after each treatment and placed into biohazard red bags to take into an incinerator.<br><br>SPAG-2 had constant crystallization of large bore tubing requiring a minimum of Q1 tubing checks to ensure delivery of ribavirin to patient.<br><br>Pooled ribavirin crystals observed on the table with nebulizer, on the floor underneath the nebulizer and tubing, and in the patient bed.<br><br>Requires the use of 7-10 liters of 100% oxygen to the patient.<br><br>Unreliable nebulization (consistently drug remains in the reservoir flask after 12 hours of continuous nebulization)	Maximum nebulization rate of 12-14ml/hour which limits the dosage and concentration options for ribavirin delivery.<br><br>Prone to crystallization in the nebulizer chamber which can block all delivery of drug to the patient.
